# A prognostic model of acute-on-chronic liver failure based on sarcopenia

**DOI:** 10.1007/s12072-022-10363-2

**Published:** 2022-06-30

**Authors:** Hong Peng, Qian Zhang, Lei Luo, Siyi Lei, Tingting Xiong, Li Long, Yan Xiong, Liulu Zhang, Jinding Zheng, Xinhua Luo

**Affiliations:** 1 Department of Infectious Diseases, Guizhou Provincial People’s Hospital, 83 Zhongshan Road, Nanming District, Guiyang, 550002 Guizhou China; 2 Good Clinical Practice Center, Guizhou Provincial People’s Hospital, Guiyang, Guizhou China

**Keywords:** Acute-on-chronic liver failure, Predictive model, Nutritional status, Sarcopenia, Liver transplantation, Survival, Progression

## Abstract

**Background:**

Acute-on-chronic liver failure (ACLF) is characterized by the development of a syndrome associated with a high risk of short-term death in patients with acute decompensated cirrhosis, and better indicators are needed to predict such outcomes. Sarcopenia, a common complication of cirrhosis, is closely associated with poor prognosis and increased mortality. In this study, the skeletal muscle index of ACLF patients was measured to determine whether sarcopenia combined with clinical parameters can aid in identifying those at high risk of progression.

**Methods:**

A total of 433 hospitalized patients with ACLF according to the APASL criteria were included and allocated into two groups: transplantation-free survival (*n* = 293) or progression (*n* = 140, 107 died; 33 underwent liver transplantation) within 90 days. Muscle mass was assessed based on the skeletal muscle index. The optimal cut-off value of the AMPAS1 model (age, MELD score, platelet count, alpha-fetoprotein level, sarcopenia, and more than one complication combination) for progression prediction was identified using receiver-operating characteristic (ROC) analysis.

**Results:**

Sarcopenia was an independent risk factor for progression in the ACLF population (HR 3.771 95% CI 2.114–6.727, *p* < 0.001). AMPAS1 was a good predictor, with an area under the ROC curve of 0.865, and the cut-off value for poor outcome prediction was 0.31 (sensitivity 79.4%, specificity 76.4%).

**Conclusion:**

We demonstrate that sarcopenia is a simple and objective indicator for predicting short-term prognosis in patients with ACLF. Moreover, compared to conventional prognostic scores, AMPAS1 is a better model for predicting 90 day adverse outcomes in ACLF patients.

**Supplementary Information:**

The online version contains supplementary material available at 10.1007/s12072-022-10363-2.

## Introduction

Acute-on-chronic liver failure (ACLF) involves rapid deterioration of liver function in chronic liver disease, and is often associated with the development of serious complications such as hepatorenal syndrome and hepatic encephalopathy (HE) within a short period of time [1, 2]. ACLF has a high rate of short-term mortality, with 28- and 90-day mortality rates as high as 25% and 40%, respectively [3]. Liver transplantation may be the only curative treatment for these patients. Although prognostic scores including Model for End-stage Liver Disease [MELD] and MELD-Na scores help guide donor liver allocation for transplantation, both of these scoring systems lack important parameters that reflect the nutritional and functional status of patients with ACLF. Indeed, the MELD score did not capture ACLF severity among candidates listed for liver transplant (LT) in a large cohort study [4]. Another multicenter study of 18,979 patients with ACLF in the United States found that MELD-Na did not capture 90 day mortality risk in ACLF, with only a small proportion of ACLF patients exceeding the median MELD thresholds for transplantation in designated LT centers [5].

To help clinicians predict the condition of patients and take timely intervention measures, a clinical index closely related to the prognosis of ACLF patients and a better prediction model are needed. Sarcopenia, a common complication of cirrhosis, is defined as a progressive and generalized loss of skeletal muscle mass, strength, and function. A recent study found that sarcopenia is strongly and independently associated with a high risk of mortality in patients with cirrhosis [6]. However, there are few real-world studies of sarcopenia in predicting the prognosis of ACLF. Therefore, in this study, a new model was developed to predict 90 day progressive risk in patients with ACLF by measuring the L3 skeletal muscle index in combination with other key clinical indicators.

## Methods

### Patients

For this retrospective study, we recruited all patients with ACLF according to the APASL criteria [chronic liver disease/cirrhosis (previously diagnosed/undiagnosed), characterized by jaundice (serum total bilirubin (TBIL) ≥ 5 mg/dl) and coagulation disorder (international normalized ratio, INR ≥ 1.5 or PTA < 40%), with concurrent ascites and/or hepatic encephalopathy within 4 weeks [7]] and treated at our institution between July 2019 and March 2021. We excluded patients younger than 18 years old, those who were pregnant, and those diagnosed with hepatocellular carcinoma or any extrahepatic malignancy, as well as patients with comorbidities associated with poor outcomes (severe cardiopulmonary disease defined by a New York Heart Association score > 3, oxygen/steroid-dependent chronic obstructive pulmonary disease, and chronic kidney disease). All patients were given standard medical treatments, including energy supplements, intravenous infusion of albumin and plasma, and preventive treatment for complications after admission. Of the 433 patients in our study, 244 (56.4%) were treated with artificial liver support. These patients were mainly in the early or middle stages of ACLF, with a PTA between 20 and 40%, or waiting for a liver donor. We mainly adopted the technique of plasma exchange with a double plasma molecule adsorption system, which, on the one hand, can supplement coagulation factors, albumin, and other substances to improve the coagulation state and, on the other hand, can completely eliminate medium- and large-molecule toxins and protein-bound toxoids and reduce plasma consumption. The 90-day transplantation-free survival rate was also determined.

The present study was approved by the ethics committee of Guizhou Provincial People’s Hospital and performed according to the ethical guidelines of the 1975 Declaration of Helsinki. The requirement for obtaining informed consent from patients was waived because of the retrospective nature of the study.

### Data collection

Demographic data and laboratory parameters for all patients were extracted through retrospective review of medical records. Laboratory parameters included prealbumin, albumin, alanine aminotransferase, aspartate aminotransferase, creatinine, TBIL, INR, white blood cell count, platelet count, and blood urea nitrogen. Effective hepatic blood flow (EHBF) was measured by the indocyanine green (ICG) clearance test to evaluate liver reserve function. Prognostic scores (including Model for End-stage Liver Disease [MELD] and MELD-Na scores) were calculated using baseline values of relevant parameters (measured at the time of admission).

### Evaluation of skeletal muscle mass and definition of sarcopenia

Skeletal muscle mass was evaluated according to the skeletal muscle index (SMI) based on computer tomography (CT) scans at L3 [8]. SMI was calculated by normalizing the L3 skeletal muscle areas by the square of the patient’s height (m^2^) [9]. The muscle masses evaluated in the L3 region were the psoas, erector spinae, quadratus lumborum, transversus abdominis, external and internal obliques, and rectus abdominis. Skeletal muscle is identified and quantified by Hounsfield unit (HU) thresholds of − 29 to + 150 [10]; with these specific HU thresholds, SMI measurements are not influenced by the presence of ascites. This analysis was performed using the diagnostic software ImageJ (NIH, Bethesda, MD, USA) [11]. Sarcopenia was defined according to the Japan Society of Hepatology guidelines for sarcopenia in liver disease as L3 SMI < 38 cm^2^/m^2^ for female patients and < 42 cm^2^/m^2^ for male patients [12].

### Statistical analysis

Continuous variables were analyzed using Student’s *t* test or the Mann–Whitney *U* test, as appropriate; the results are expressed as the mean ± standard deviation or median (25th centile; 75th centile). Categorical data were compared using the Chi-square test, and the results are expressed as numbers (percentages). Cox proportional hazard model and receiver-operating characteristic curve (ROC) analyses were used to identify independent factors for progression in patients with ACLF. Then, we compared the area under the curves (AUC) using the DeLong test. Finally, the independent predictors obtained from the above screening progress were considered for nomogram construction. Calibration curves and the C-index were subsequently evaluated to assess the calibration of the model. Cumulative rates of the 90 day transplantation-free survival rate were plotted using a Kaplan–Meier curve and compared using the log-rank test. All statistical analyses were carried out using SPSS 25.0 (Chicago, IL, USA) and R 3.6.0. Statistical significance was set at two-sided *p* < 0.05.

## Results

### Patient characteristic

Of the 523 patients who were recruited for the present study, 90 were excluded: 42 due to hepatocellular carcinoma, 21 due to other extrahepatic malignancies, 7 due to pregnancy, 5 due to severe cardiopulmonary disease, 3 due to chronic obstructive pulmonary disease, and 12 due to chronic kidney disease. Ultimately, 433 patients with ACLF were enrolled for analysis. In this cohort, the duration of the disease was 47.8 ± 6.9 days, and the duration of hospital stay was 14.7 ± 8.3 days. The baseline characteristics and biochemical data of the participants are listed in (Table 1). Among the 433 participants, 293 were in the transplantation-free survival group, and 140 were in the progression group (107 died; 33 underwent liver transplantation), representing a 90 day transplantation-free survival rate of 67.7%. Overall, mean age, MELD score, lymphocyte count, NLR, TBIL, INR, CRP, IL-6, proportion of sarcopenia, and more than one complication were higher in the patients who experienced progression; conversely, the mean L3 SMI, platelet count, ALT, sodium, and AFP were lower (*p* < 0.05). At the same time, we compared 28 day transplantation-free survival in the 433 patients, with 377 (87.1%) having transplantation-free survival. The mean age, MELD score, TBIL, INR, and proportions of patients with sarcopenia and more than one complication were higher in the progression group, and the increase in IL-6 was close to statistically significant (Table S1).

**Table 1:** Baseline characteristics of the study participants

Characteristic	Total (*N* = 433)	90 Day transplantation-free survival (*n* = 293)	Progression (*n* = 140)	*p* Value
Age, y	47.0 (34.8–54.3)	47.0 (38.0–54.0)	49.0 (45.0–63.0)	0.006
Sex, *n* (%)				0.462
Male	346 (79.90)	237 (80.90)	109 (77.90)	
Female	87 (20.10)	56 (19.10)	31 (22.10)	
MELD score	22.0 (16.8–25.0)	22.0 (17.0–24.0)	21.0 (18.0–27.0)	< 0.001
Etiology, * n* (%)				0.541
HBV/HCV	232 (53.60)	154 (52.60)	78 (55.70)	
Alcohol	100 (23.10)	73 (24.90)	27 (19.30)	
HBV/HCV + Alcohol	36 (8.30)	25 (8.50)	11 (7.90)	
Others	65 (15.00)	41 (14.00)	24 (17.10)	
L3 SMI, cm^2^/m^2^	40 (36–45.5)	41.1 (34.3–45.5)	36.0 (30.0–38.9)	< 0.001
Sarcopenia, *n* (%)	250 (57.7)	134 (45.7)	116 (82.9)	< 0.001
BMI	23.7 (21.6–26.4)	23.8 (21.7–27.2)	23.6 (21.5–26.2)	0.989
Platelet (× 10^9^/L)	88.5 (60.8–131.0)	101.0 (66.0–151.0)	64.0 (43.0–99.0)	< 0.001
Neutrophil (× 10^9^/L)	2.95 (1.26–5.44)	4.06 (2.05–6.80)	3.45 (0.89–4.78)	0.435
Lymphocyte (× 10^9^/L)	1.45 (0.85–4.02)	1.22 (0.76–1.87)	1.07 (0.68–3.37)	0.016
NLR	2.21 (0.26–6.03)	3.48 (1.82–7.42)	3.93 (0.19–8.39)	0.019
PLR	57.88 (17.40–101.52)	87.03 (47.24–135.07)	59.53 (13.06–121.25)	0.955
Serum albumin, g/L	28.1 (25.8–31.3)	28.3 (25.7–32.4)	26.0 (21.1–28.3)	0.31
Creatinine, µmol/L	71.0 (60.0–88.0)	69.5 (57.1–78.5)	88.9 (64.8–126.3)	0.068
Total bilirubin, µmol/L	244.6 (161.7–332.5)	255.5 (164.3–325.4)	280.9 (208.2–491.8)	< 0.001
Alanine aminotransferase, U/L	112.5 (36.5–522.3)	112.5 (38.0–485.0)	54.5 (27.5–138.8)	0.033
International normalized ratio	1.82 (1.56–2.09)	1.75 (1.56–2.08)	1.90 (1.65–2.46)	< 0.001
C-reactive protein, mg/L	12.06 (7.27–28.25)	10.73 (6.28–23.73)	27.08 (12.96–47.81)	0.002
Interleukin-6, pg/mL	18.34 (8.55–32.46)	18.04 (8.95–30.52)	49.62 (23.26–88.48)	0.002
Effective hepatic blood flow, L/min	0.20 (0.14–0.27)	0.22 (0.17–0.30)	0.17 (0.11–0.20)	0.063
Sodium, mmol/L	136.0 (133.0–138.4)	137.0 (134.0–138.0)	133.0 (131.0–137.0)	0.009
Prealbumin, g/L	59.9 (50–89.2)	60.7 (50–89.7)	69.9 (50.8–91.1)	0.806
Alpha-fetoprotein, mmol/L	28.0 (5.0–119.6)	29.2 (5.5–108.7)	5.2 (2.86–34.4)	0.006
^a^More than one complication, *n* (%)	223 (51.5)	110 (37.5)	113 (80.7)	< 0.001

Continuous variables are expressed as the median (IQR)

*MELD score* end-stage liver disease score, *HBV* hepatitis B virus, *HCV* hepatitis C virus, *L3 SMI* L3 skeletal muscle index, *BMI* body mass index, *NLR* neutrophil-to-lymphocyte ratio, *PLR* platelet-to-lymphocyte ratio

^a^More than one complication included gastroesophageal varices, hepatic encephalopathy, acute kidney injury, and infections

### Factors associated with progression

Univariate analysis revealed sarcopenia, more than one complication (including gastroesophageal varices, hepatic encephalopathy, acute kidney injury, and infection), age, MELD score, CRP ≥ 20 mg/L, sodium < 135 mmol/L, platelet < 100 × 10^9^/L and AFP < 6.2 mmol/L to be associated with 90 day progression in patients with ACLF (cut-off values of CRP, platelet, and AFP were analyzed by ROC curve analysis). However, multivariate analysis identified only sarcopenia, more than one complication, age, MELD score, platelet < 100 × 10^9^/L, and AFP < 6.2 mmol/L as independent predictors of progression (Table 2). In addition, we calculated the C-index to be 0.804, which indicated that the model had a high degree of differentiation and accuracy. We also compared independent risk factors in patients with progression at 28 days, but only age, MELD score, and CRP were found to be independent predictors by multivariate analysis (Table S2).

**Table 2:** Univariate and multivariate Cox proportional hazard models to predict 90 day progression in ACLF patients

Variables	Univariate analysis			Multivariate analysis		
	HR	95% CI	*p* Value	HR	95% CI	*p* Value
Age > 60 y	1.738	1.189–2.539	0.004	1.728	1.083–2.757	0.022
MELD score > 20	2.321	1.557–3.460	< 0.001	2.326	1.431–3.783	0.001
C-reactive protein > 20, mg/L	1.776	1.260–2.502	0.001			
Sodium < 135 mmol/L	1.977	1.417–2.757	< 0.001			
Platelet < 100 × 10^9^/L	1.623	1.144–2.302	0.007	1.825	1.117–2.983	0.016
Alpha-fetoprotein < 6.2 mmol/L	1.799	1.271–2.546	0.001	2.219	1.442–3.417	< 0.001
Sarcopenia	4.119	2.505–6.771	< 0.001	3.771	2.114–6.727	< 0.001
aMore than one complication	5.033	3.305–7.665	< 0.001	3.888	2.303–6.562	< 0.001

*HR* hazard ratio, *CI* confidence interval, *MELD* score, end-stage liver disease score

^a^More than one complication included gastroesophageal varices, hepatic encephalopathy, acute kidney injury, and infections

### Analysis of the predictive value of the model

**Fig. 1:**
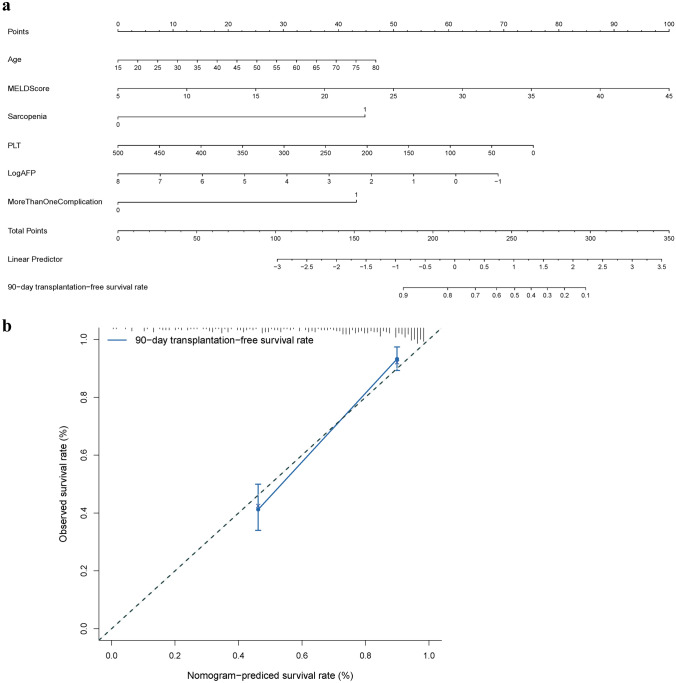
Nomogram to predict the probability of 90 day transplantation-free survival in patients with acute-on-chronic liver failure. Calibration curve to predict the probability of 90 day transplantation-free survival

Next, we built a nomogram by combining prognostic factors, including age, MELD score, platelet count, AFP, sarcopenia, and more than one complication (Fig. 1a), and the vertical line from the coordinate axis of each risk index was used to obtain corresponding scores. These scores were added to obtain the total score, corresponding to the 90 day transplantation-free survival rate in the last coordinate axis, and the survival percentage for each individual was obtained. To assess our model, we drew a calibration curve based on the actual incidence and the prediction rate, which showed that the apparent curve had a similar prediction function compared with the ideal model (Fig. 1b).

**Fig. 2:**
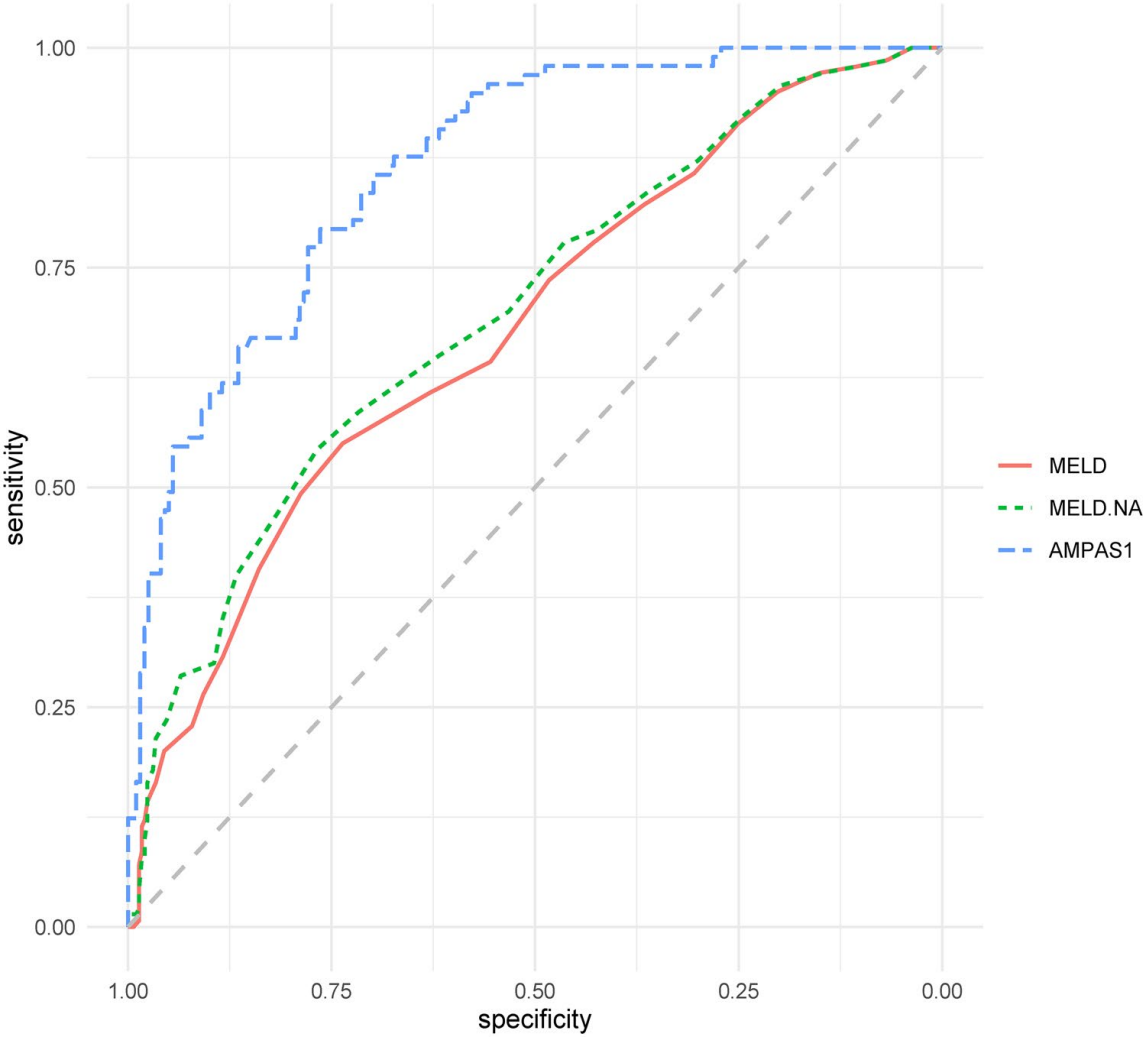
Receiver-operating characteristic curves indicating the relative efficiencies of the Chinese cohort with acute-on-chronic liver failure of end-stage liver disease (MELD) score. End-stage liver disease includes serum sodium (MELD-Na) and the combination of age, MELD score, platelet count, AFP, sarcopenia, and more than one complication (AMPAS1) for predicting 90-day progression in patients with acute-on-chronic liver failure

Furthermore, the prognostic value of the MELD score and MELD-Na score for predicting outcomes was assessed by analyzing the area under the ROC curve. The sensitivity and specificity were 82.1 and 36.6% for the MELD score and 58.6 and 71.7% for the MELD-Na score, respectively. The powers of the MELD score and MELD-Na score for predicting outcome were not significantly different, as indicated by their similar area under the curve values (0.680 and 0.723, respectively, *p* = 0.076). When age, MELD score, platelet count, AFP, sarcopenia, and more than one complication were combined (AMPAS1), the area under the curve for predicting mortality was 0.865, which was higher than that of either parameter alone (both *p* < 0.001), and the cut-off value, sensitivity, and specificity were 0.31, 79.4, and 76.4% (Fig. 2).

### Two risk groups for prediction of 90 day progression

**Fig. 3:**
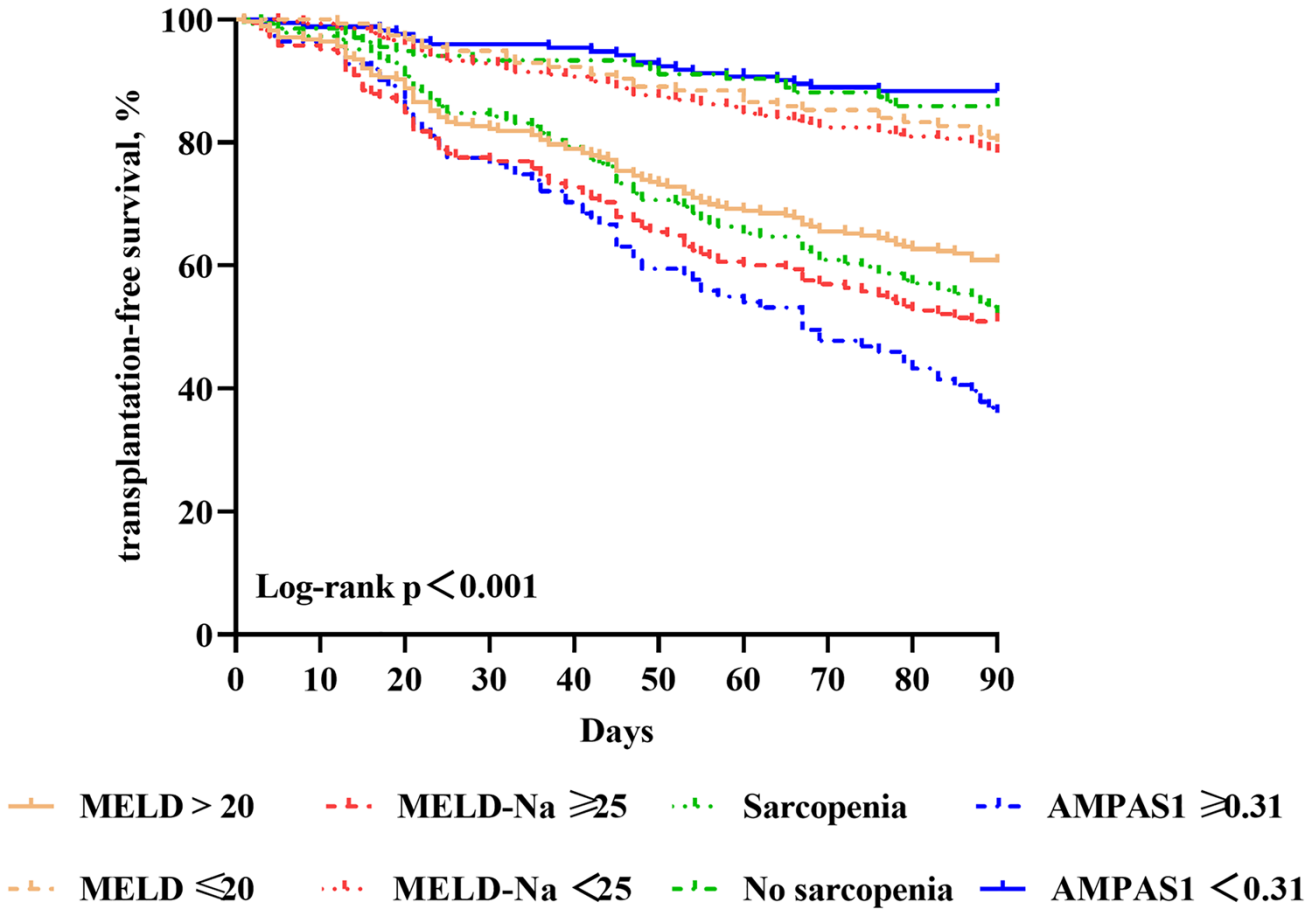
Kaplan–Meier curve depicting 90 day transplantation-free survival in patients with acute-on-chronic liver failure

Finally, patients were divided into two significantly different risk groups (high and low) according to the preselected cut-off points, and 90 day progression in these patients was compared. According to previous clinical studies, patients with MELD scores > 20 and MELD-Na scores ≥ 25 are considered to be at high risk, [13–15] and a total of 276 and 165 ACLF patients in our study met these criteria. In addition, the cut-off value of AMPAS1 was 0.31. In total, 124 high-risk patients were screened by ROC analysis; according to Cox proportional hazard regression, patients in the high-risk group with MELD scores > 20 had a 2.3-fold higher likelihood of progressive events than did patients in the low-risk group with MELD scores below 20, and those in the high-risk group with MELD-Na scores had a 2.9-fold higher likelihood of progression than did low-risk patients with MELD-Na scores below 25. Moreover, those with sarcopenia had a 3.1-fold higher likelihood of progression than did those without sarcopenia, and AMPAS1 ≥ 0.31 in the high-risk group was associated with a 7.5-fold higher progressive event likelihood than low-risk patients with AMPAS1 below 0.31 (Table 3). Moreover, 90 day cumulative survival rates were compared using the different models between the low- and high-risk groups. Survival for patients with AMPAS1 ≥ 0.31 was extremely low, at 36.0%; survival for those with sarcopenia, MELD-Na and MELD was 52.2, 50.9 and 60.5%, respectively. Similarly, the median survival 61.1 days based on AMPAS1 (95% CI (55.7–66.4)), 68.6 days based on sarcopenia (95% CI (64.5–72.7)), 64.1 days based on MELD-Na (95% CI (59.3–68.9)), and 69.8 days based on MELD (95% CI (66.4–73.3)). There was a significant difference in survival curves among the groups (*p* < 0.001) (Fig. 3).

**Table 3:** Risk of 90 day progressive events for two risk groups defined by test-specific cutoffs

Risk groups	Event (%)	Hazard ration	*p* value
MELD score			
≤ 20	31 (22.10)	1	–
> 20	109 (40.20)	2.321 (1.557–3.460)	< 0.001
MELD-Na score			
< 25	58 (41.40)	1	-
≥ 25	82 (58.60)	2.876 (2.053–4.028)	< 0.001
Sarcopenia			
No	25 (17.90)	1	–
Yes	115 (82.10)	3.119 (2.505–6.771)	< 0.001
AMPAS1			
< 0.31	29 (20.6)	1	–
≥ 0.31	111 (79.4)	7.523 (4.591–12.327)	< 0.001

Hazard ratios from univariable Cox proportional hazard regression for prediction of 90 day progressive events according to low- and high-risk patients, with *P* values for between-group differences shown as hazard ratios. We used previously published cut-off points to define the risk groups

MELD score, End-stage Liver Disease score; MELD-Na, End-stage Liver Disease includes serum sodium score; AMPAS1, including age, MELD score, platelet, AFP, sarcopenia, and more than one complication

## Discussion

Differences between Eastern and Western diagnostic criteria for ACLF have resulted in considerable discrepancies in the identification, rescue regimen, and eventual prognosis of the condition [16–18]. The main causes of liver failure in Western countries are non-alcoholic steatohepatitis and alcohol consumption; in Asian, the main cause is hepatitis virus infection, especially hepatitis B virus [19]. The established MELD score and MELD-Na score prognostic models are based on European and American studies, and better predictive models for Asian patients with ACLF need to be further explored. For the present study, we used the Asian criteria for ACLF, which have been validated for the diagnosis of ACLF in China. Our analyses of a cohort of patients with ACLF revealed three major findings. First, to our knowledge, this is the first study to report sarcopenia as an independent risk factor for progression in an ACLF population. Second, we are the first to combine sarcopenia with clinical indicators to predict poor 90 day outcomes in patients with ACLF. Third, MELD scores and MELD-Na scores did not capture the severity of 90 day adverse outcomes in patients with ACLF, whereas our new model, AMPAS1, was a good predictor of such outcomes. Interestingly, by comparing clinical markers of patients with 28 day progression to those with 90 day progression, we also found that sarcopenia was not an independent risk factor for 28 day progression. We suspect that the reason for this difference may be related to the different mechanisms of disease progression. Patients who experienced progression at 28 days were more likely to have an acute inflammatory storm with massive hepatocyte necrosis, whereas progression at 90 days may be associated with anabolism and even host immune function.

The MELD score is the most commonly used model to predict the prognosis of liver disease and incorporates three laboratory variables: TBIL, INR, and creatinine [13, 14]. Although the MELD score considers liver dysfunction and renal insufficiency, it does not incorporate other crucial factors, such as HE, organ failures, or infection, which can affect prognosis [20]. Moreover, our data show that both the MELD score and MELD-Na score do not fully capture underlying disease severity in ACLF. This is likely due to the effect of extrahepatic organ failures on patient outcomes, which are not considered when calculating MELD-Na. Scores other than MELD or MELD-Na based on organ failure may be more appropriate to predict prognosis in patients with ACLF. Previous studies have identified an association between several factors, including age, AFP, and platelets, and poor outcomes related to liver failure, which is consistent with our study [21]. In addition, the nutritional status of patients with liver disease is a problem that is easy to ignore. Sarcopenia has also been found to be associated with high mortality and poor posttransplant outcomes in patients awaiting liver transplantation [22–24]. The present study complements these studies, adding sarcopenia, complications and clinical indicators as a predictor of prognosis in patients with ACLF, and combining these elements with the MELD score adds to the power of the score for predicting progression.

The relationship between sarcopenia and unfavorable outcomes of ACLF is complex and has yet to be fully elucidated. Because ACLF itself is associated with mortality, systemic inflammation, and organ failure, it is unclear which factors associated with sarcopenia are relevant for ACLF. The present study indicates that sarcopenia is an independent risk factor for progression in the ACLF population, as patients presenting sarcopenia had a higher risk of LT or mortality than those without sarcopenia. Our conclusions are consistent with those of Montano-Loza and colleagues, [25] who determined that sarcopenia is associated with a fivefold increased risk of mortality in patients with cirrhosis, independent of the MELD-Na score. Tandon et al. also reported that sarcopenia is associated with a twofold increased risk of liver transplant waitlist mortality [26]. Sarcopenia results from an imbalance between protein synthesis and catabolism. Major pathways involved in peripheral muscle breakdown include activation of autophagy and the ubiquitin–proteasome pathway (UPP) and the mammalian target of rapamycin (mTOR) pathway [27, 28]. Myostatin, a myokine, is an inhibitor of protein synthesis and regeneration. In addition, a number of proinflammatory cytokines (TNF-α, IL-6, IL-1 and IFN-γ) have been reported to trigger muscle wasting, but the precise contributions of these factors remain controversial and unclear under different conditions [27]. Hyperammonemia, which upregulates myostatin levels and activates autophagy, also plays a crucial role in the development of sarcopenia [29]. Elevated levels of ammonia lead to impairment of the mTOR pathway through increased mitochondrial dysfunction and production of reactive oxygen species (ROS) [29]. We hypothesize that the progression of ACLF and sarcopenia are causally related and that the release of large amounts of inflammatory factors and the further elevation of blood ammonia during the course of ACLF inhibits muscle synthesis and regeneration. At the same time, muscle anabolic dysfunction and increased catabolism may further promote the progression of the disease in patients with ACLF. Consequently, malnutrition has been shown to lead to increased liver damage and worse outcomes (30).

Our study also has some limitations. First, the study was retrospective in nature, and the sample size was small. Whether these results are generalizable to patients with ACLF defined according to the Western criteria (CLIF) remains unknown. Meanwhile, due to the limitations of the retrospective study, it was impossible to quantify the nutritional intake of these patients during their hospital stay. Second, L3 SMI levels were not measured dynamically; thus, it remains unclear whether dynamic changes in L3 SMI levels have more clinical value in predicting adverse outcomes in patients with ACLF. Third, the mechanism underlying the contribution of sarcopenia to the progression of ACLF remains unknown. Further prospective multicenter studies are needed to clarify these uncertainties and confirm our conclusions.

In summary, the current study indicates that sarcopenia is a simple and objective indicator that can predict the 90 day prognosis of patients with ACLF. Nutritional status may provide valuable information to supplement the conventional approaches of assessing disease condition in these patients, representing a useful tool in clinical practice to assess patient prognosis and help clinicians identify individuals in need of nutritional intervention. AMPAS1 is a better model to predict 90 day adverse outcomes in patients with ACLF than the conventional prognostic scores.

## Supplementary Information

Below is the link to the electronic supplementary material.

Supplementary file1 (DOCX 18 KB)

## Data Availability

The data that support the findings of this study are available from the corresponding author upon reasonable request.
