# Supplementary material for: A prognostic model of acute-on-chronic liver failure based on sarcopenia
Source: Hepatol Int. 2022 Jun 30;16(4):964–72. doi: 10.1007/s12072-022-10363-2 (PMC9349113; doi:10.1007/s12072-022-10363-2)
Supplement: Supplementary file 1 — Supplementary file1 (DOCX 18 KB) [file 12072_2022_10363_MOESM1_ESM.docx]

Table S1 Baseline characteristics of the study participants

| **Characteristic** | **Total (N = 433)** | **28-day survival (N=377)** | **Non-survival (N=56)** | **p** |
| --- | --- | --- | --- | --- |
| Age, y | 47.0 (38.0-54.0) | 47.0 (38.0-54.0) | 50.0 (45.0-66.0) | 0.021 |
| Sex, n (%) |  |  |  | 0.928 |
| Male | 346 (79.9) | 301 (79.8) | 45 (80.4) |  |
| Female | 87 (20.1) | 76 (20.2) | 11 (19.6) |  |
| MELD score | 22.0 (18.0-25.0) | 22.0 (18.0-25.0) | 25.0 (18.0-29.0) | <0.001 |
| Etiology, n (%) |  |  |  | 0.651 |
| HBV/HCV | 232 (53.6) | 198 (52.5) | 34 (60.7) |  |
| Alcohol | 100 (23.1) | 88 (23.3) | 12 (21.4) |  |
| HBV/HCV+Alcohol | 24 (7.60) | 22 (8.10) | 2 (4.80) |  |
| Others | 65 (15.0) | 58 (15.4) | 7 (12.5) |  |
| L3 SMI, cm^2^/m^2^ | 39.3 (32.6-44.1) | 39.4 (33.6-44.1) | 28.2 (26.7-43.0) | 0.004 |
| Sarcopenia, n (%) | 250 (57.7) | 208 (55.3) | 42 (75.7) | 0.018 |
| BMI | 23.8 (21.6-26.5) | 23.8 (21.6-26.6) | 22.4 (21.6-25.9) | 0.934 |
| Platelet (×10^9^/L) | 95.0 (63.0-141.0) | 95.0 (63.0-144.0) | 43.0 (28.0-96.0) | 0.178 |
| Neutrophil (×10^9^/L) | 4.04 (1.91-6.54) | 4.05 (1.95-6.68) | 4.03 (0.57-4.47) | 0.999 |
| Lymphocyte (×10^9^/L) | 1.20 (0.76-1.88) | 1.20 (0.77-1.87) | 0.67 (0.25-2.99) | 0.789 |
| NLR | 3.52 (1.44-7.64) | 3.52 (1.64-7.26) | 7.63 (0.19-22.03) | 0.513 |
| PLR | 79.97 (42.83-129.19) | 76.61 (43.78-129.21) | 101.49 (14.23-260.26) | 0.577 |
| Serum albumin, g/L | 27.9 (25.4-30.9) | 27.9 (25.5-31.0) | 25.8 (18.5-29.3) | 0.112 |
| Creatinine, µmol/L | 71.0 (60.0-88.0) | 71.0 (60.0-87.5) | 87.0 (70.7-163.9) | 0.125 |
| Total bilirubin, µmol/L | 264.6 (173.5-341.4) | 264.6 (170.4-339.9) | 438.4 (245.4-496.7) | <0.001 |
| Alanine aminotransferase, U/L | 90.5 (35.8-453.0) | 91.0 (35.0-474.5) | 53.0 (36.5-130.5) | 0.156 |
| International normalized ratio | 1.79 (1.58-2.12) | 1.78 (1.58-2.18) | 1.91 (1.77-1.96) | <0.001 |
| C-reactive protein, mg/L | 11.41 (7.06-27.62) | 11.28 (6.99-27.41) | 49.52 (22.97-111.35) | 0.135 |
| Interleukin-6, pg/mL | 20.65 (11.20-46.12) | 20.28 (10.10-41.76) | 24.93 (13.95-91.11) | 0.051 |
| Effective hepatic blood flow, L/min | 0.21 (0.16-0.29) | 0.21 (0.16-0.29) | 0.17 (0.06-0.19) | 0.024 |
| Sodium, mmol/L | 136.0 (133.0-138.1) | 136.0 (133.0-138.2) | 133.0 (128.7-136.3) | 0.062 |
| Prealbumin, g/L | 61.9 (50.0-90.0) | 61.6 (50.0-90.0) | 67.0 (50.0-86.9) | 0.224 |
| Alpha-fetoprotein, mmol/L | 23.5 (4.7-103.0) | 25.13 (4.77-104.45) | 5.42 (3.53-29.95) | 0.867 |
| ^*^More than one complication, n (%) | 223 (51.5) | 179 (47.5) | 44 (78.6) | <0.001 |

Continuous variables are expressed as the median (IQR).

Abbreviations: MELD score, end-stage liver disease score; HBV, hepatitis B virus; HCV, hepatitis C virus; L3 SMI, L3 skeletal muscle index; BMI, body mass index; NLR, neutrophil-to-lymphocyte ratio; PLR, platelet-to-lymphocyte ratio.

^*^More than one complication included gastroesophageal varices, hepatic encephalopathy, acute kidney injury and infections.

Table S2 Univariate and multivariate Cox proportional hazard models to predict 28-day progression in ACLF patients

| **Variables** | **Univariate analysis** | | | **Multivariate analysis** | | |
| --- | --- | --- | --- | --- | --- | --- |
|  | **HR** | **95% CI** | **p** | **HR** | **95% CI** | **p** |
| Age | 1.027 | 1.006-1.049 | 0.013 | 1.051 | 1.022-1.081 | <0.001 |
| MELD score | 1.078 | 1.040-1.118 | <0.001 |  |  |  |
| C-reactive protein | 1.003 | 1.001-1.005 | 0.004 | 1.003 | 1.000-1.005 | 0.023 |
| Sarcopenia | 2.358 | 1.113-4.998 | 0.025 |  |  |  |
| *More than one complication | 3.766 | 1.989-7.132 | <0.001 | 2.491 | 1.160-5.352 | 0.019 |

Abbreviations: HR, hazard ratio; CI, confidence interval; MELD score, end-stage liver disease score; NLR, neutrophil-to-lymphocyte ratio.

^*^More than one complication included gastroesophageal varices, hepatic encephalopathy, acute kidney injury and infections.
